# Supplementary material for: Microbial Diversity and Volatile Flavor Compounds in Tibetan Flavor Daqu
Source: Foods. 2023 Jan 9;12(2):324. doi: 10.3390/foods12020324 (PMC9858000; doi:10.3390/foods12020324)
Supplement: Supplementary file 1 [file foods-12-00324-s001.zip › foods-2062287-supplementary.pdf]

Supplementary Materials

Microbial Diversity and Volatile Flavor Compounds in Tibetan Flavor *Daqu*

Yaping Li [1], Haijun Qiao [2], Rui Zhang [1], Weibing Zhang [1,*] and Pengcheng Wen [3,*]

[1] College of Food Science and Engineering, Gansu Agricultural University, Lanzhou 730070, China
[2] College of Science, Gansu Agricultural University, Lanzhou 730070, China
[3] Functional Dairy Product Engineering Lab of Gansu Province, Yingmen Village, Anning, Lanzhou, Gansu 730070, China
* Correspondence: zzz888666@gsau.edu.cn (W.Z.); wenpch@126.com (P.W.)

Table S1. Species statistics by class of samples.

	Sample	Phylum	Class	Order	Family	Genus	Species
Bacteria	ZQ1	14	24	52	65	83	87
	ZQ2	7	9	16	22	30	36
	ZQ3	6	7	14	19	28	36
	ZQ4	8	12	23	31	39	44
	ZQ5	12	23	45	64	75	78
	Total	16	39	85	118	158	171
Fungi	ZQ1	3	7	7	11	12	13
	ZQ2	3	5	5	9	10	10
	ZQ3	3	5	5	10	12	13
	ZQ4	3	5	5	10	12	13
	ZQ5	5	10	14	21	29	30
	Total	5	11	19	27	35	39

Table S2. Differences in the dominant microorganisms of different types of Daqu.

Types of Daqu	Dominant Bacteria	Dominant Fungi	Detection method
Tibetan flavor Daqu	*Oceanobacillus, Kroppenstedtia, Virgibacillus, Enterococcus, Pediococcus, Streptomyces,Saccharopolyspora, Leuconostoc, uncultured_bacterium_f_ Lachnospiraceae, Lactobacillus*	*Wickerhamomyces, Monascus, Aspergillus, Rhizomucor*	Single molecule real-time sequencing
Jiang-flavorDaqu	*Bacillales, Enterobacteriales, Lactobacillales*	*Candida, Trichoderma, Aspergillus, Trichosporon, Thermomyces*	Illumina MiSeq

strong-flavor Daqu	*Lactobacillus, Aquabacterium, Thermoactinomyces*	*Aspergillus, Thermoascus, Kazachstania, Pichia, and Rhizomucor*	High-throughput sequencing
Light-flavor Daqu	*Kroppenstedtia, Oceanobacillus, Lactobacillus, Paucisalibacillus, Bacillus, Lentibacillus*	*Aspergillus, Pichia, Saccharomycopsis*	High-throughput sequencing
Te-flavour Daqu	*Oceanobacillus, Kroppenstedtia, Pediococcus, Weissella, Bacillus, Alcaligenes, Lactobacillus, Saccharopolyspora, Enterococcus*	*Aspergillus, Thermoascus, Pichia, Saccharomycopsis, Sclerotinia, Millerozyma*	Illumina MiSeq
